# 5,9-Dihy­droxy-9-methyl-3,6-dimethyl­ene-3a,4,5,6,6a,7,8,9,9a,9b-deca­hydro­azuleno[4,5-*b*]furan-2(3*H*)-one

**DOI:** 10.1107/S160053681102352X

**Published:** 2011-06-22

**Authors:** Mohamed Moumou, Ahmed Benharref, Moha Berraho, Lahcen El Ammari, Mohamed Akssira, Ahmed Elhakmaoui

**Affiliations:** a Laboratoire de Chimie Biomoléculaire, Substances Naturelles et Réactivité, URAC 16, Faculté des Sciences Semlalia, BP 2390, Bd My Abdellah, 40000 Marrakech, Morocco; b Laboratoire de Chimie du Solide Appliqueé, Faculté des Sciences, Avenue Ibn, Battouta BP 1014 Rabat, Morocco; c Laboratoire de Chimie Bioorganique et Analytique, URAC 22, BP 146, FSTM, Université Hassan II, Mohammedia-Casablanca 20810 Mohammedia, Morocco

## Abstract

The title compound, C_15_H_20_O_4_, was synthesized from 9α-hy­droxy­parthenolide (9α-hy­droxy-4,8-dimethyl-12-methyl­ene-3,14-dioxatricyclo­[9.3.0.0^2,4^]tetra­dec-7-en-13-one), which was isolated from the chloro­form extract of the aerial parts of *Anvillea radiata*. The seven-membered ring has a chair conformation, while the five-membered rings display twisted conformations. The dihedral angle between the seven-membered ring and the lactone ring is 21.69 (10)°. In the crystal, mol­ecules are linked into chains propagating along the *c* axis by inter­molecular O—H⋯O hydrogen bonds; an intra­molecular O—H⋯O link also occurs.

## Related literature

For background to the medicinal uses of the plant *Anvillea radiata*, see: Abdel Sattar *et al.* (1996 ▶); Bellakhdar (1997 ▶); El Hassany *et al.* (2004 ▶); Qureshi *et al.* (1990 ▶). For the reactivity of this sesquiterpene, see: El Haib *et al.* (2011 ▶). For ring puckering parameters, see: Cremer & Pople (1975 ▶).
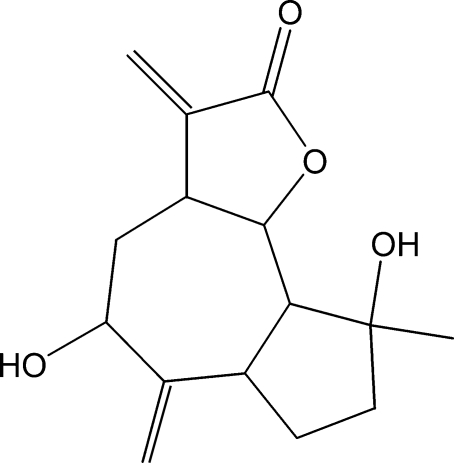


## Experimental

### 

#### Crystal data

C_15_H_20_O_4_
*M* *_r_* = 264.31
Orthorhombic, 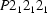

*a* = 6.4210 (14) Å
*b* = 13.504 (3) Å
*c* = 15.619 (3) Å
*V* = 1354.4 (5) Å^3^
*Z* = 4
Mo *K*α radiation
μ = 0.09 mm^−1^
*T* = 298 K
0.50 × 0.33 × 0.08 mm

#### Data collection

Bruker APEXII CCD area-detector diffractometer
14445 measured reflections
1610 independent reflections
1473 reflections with *I* > 2σ(*I*)
*R*_int_ = 0.051

#### Refinement

*R*[*F* ^2^ > 2σ(*F* ^2^)] = 0.037
*wR*(*F* ^2^) = 0.097
*S* = 1.08
1610 reflections
175 parameters
H-atom parameters constrained
Δρ_max_ = 0.20 e Å^−3^
Δρ_min_ = −0.18 e Å^−3^

### 

Data collection: *APEX2* (Bruker, 2005 ▶); cell refinement: *APEX2* and *SAINT* (Bruker, 2005 ▶); data reduction: *SAINT*; program(s) used to solve structure: *SHELXS97* (Sheldrick, 2008 ▶); program(s) used to refine structure: *SHELXL97* (Sheldrick, 2008 ▶); molecular graphics: *ORTEP-3 for Windows* (Farrugia, 1997 ▶)and *PLATON* (Spek, 2009 ▶); software used to prepare material for publication: *WinGX* (Farrugia, 1999 ▶).

## Supplementary Material

Crystal structure: contains datablock(s) I, global. DOI: 10.1107/S160053681102352X/sj5165sup1.cif

Structure factors: contains datablock(s) I. DOI: 10.1107/S160053681102352X/sj5165Isup2.hkl

Supplementary material file. DOI: 10.1107/S160053681102352X/sj5165Isup3.cml

Additional supplementary materials:  crystallographic information; 3D view; checkCIF report

## Figures and Tables

**Table 1:** Hydrogen-bond geometry (Å, °)

*D*—H⋯*A*	*D*—H	H⋯*A*	*D*⋯*A*	*D*—H⋯*A*
O2—H2⋯O3	0.82	2.42	3.015 (2)	131
O4—H4⋯O2^i^	0.82	2.03	2.819 (2)	162

Symmetry code: (i) 
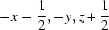
.

**Fig. 1.**
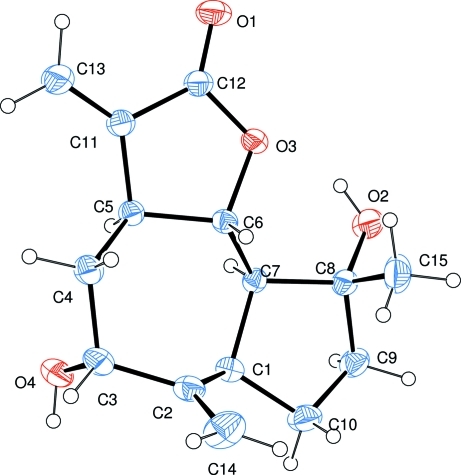
: Molecular structure of the title compound with the atom-labelling scheme. Displacement ellipsoids are drawn at the 30% probability level. H atoms are represented as small spheres of arbitrary radii.

**Fig. 2.**
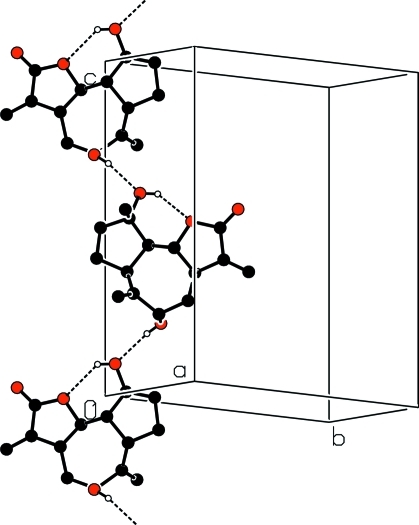
: Partial packing view showing the O –H···O interactions (dashed lines) and the formation of a chain parallel to the c axis. H atoms not involved in hydrogen bonding have been omitted for clarity. [Symmetry code: (i) -x - 1/2,-y,+z + 1/2].

## supplementary crystallographic information

### Comment

*Anvillea radiata* is a plant that grows in northern Africa and particularly in the two Maghreb countries, Morocco and Algeria. This plant is used in traditional local medicine for the treatment of dysentery, gastric-intestinal disorders (Bellakhdar, 1997), and hypoglycemic activity (Qureshi *et al.*, 1990), and has been reported to have antitumor activity (Abdel Sattar *et al.*, 1996). In our study of different Moroccan endemic plants, we have demonstrated that the aerial parts of *Anvillea radiata* could be used as a renewable source of 9-hydroxyparthenolide (El Hassany, *et al.*, 2004). In order to prepare products with high added value that can be used in the pharmacology and cosmetics industries, we have studied the chemical reactivity of this major constituent of *Anvillea radiata*.  Thus, treatment of this sesquiterpene with methane sulfonic acid (MSA) or *p*-toluene sulfonic acid (PTSA) in dichloromethane (El Haib *et al.*, 2011) led to 5,9-dihydroxy-9-methyl- 3,6-dimethylene-decahydro-azulene [4,5-*b*] furan-2-one with a yield of 45%. The molecule contains three fused rings which exhibit different conformations. The molecular structure of (I), Fig.1, shows the five membered rings to adopt a twisted conformations, as indicated by the Cremer & Pople (1975) puckering parameters Q = 0.233 (2) Å and φ = 121.1 (5)° for the lactone ring and Q = 0.426 (2) Å, φ = 264.5 (3)° for the other five-membered ring. The seven-membered ring has a chair conformation with QT = 0.8255 (20) Å, θ2 = 36.20 (15)°, φ2 = 89.3 (2)° and φ3 =207.07 (18). In the crystal structure, molecules are linked into chains (Fig. 2) running along the *c* axis by intermolecular O4—H2···O3 hydrogen bonds. In addition an intramolecular O2—H2···O3 hydrogen bond is also observed.

### Experimental

Methane sulfonic acid (MSA) or *p*-toluene sulfonic acid (PTSA) (6x10_-2_mmol) was added to a stirred solution of 9α-hydroxyparthenolide (1 g, 4 mmol) in dichloromethane (10 ml). The reaction mixture is left stirring for two hours at room temperature. After completion of the reaction, a saturated solution of NaHCO_3_ was added and the resulting mixture is extracted three times (3x20mL) with dichloromethane. The organic phases are combined and dried over Na_2_SO_4_ and evaporated under vacuum. Chromatography of the residue obtained on a column of silica gel eluting with hexane - ethyl acetate (40/60) allowed the isolation of pure 5,9-dihydroxy-9-methyl- 3,6-dimethylene-decahydro-azulene [4,5-*b*] furan-2-one (446 mg, 1.80 mmol). The title compound was recrystallized from its ethyl acetate solution.

### Refinement

All H atoms were fixed geometrically and treated as riding with C—H = 0.96 Å (methyl), 0.97 Å (methylene), 0.98Å (methine) with *U*_iso_(H) = 1.2*U*_eq_(methylene, methine) or *U*_iso_(H) = 1.5*U*_eq_(methyl, OH). In the absence of significant anomalous scattering, the absolute configuration could not be reliably determined and thus 1148 Friedel pairs were merged and any references to the Flack parameter were removed.

### Crystal data

**Table:**

C_15_H_20_O_4_	*F*(000) = 568
*M**_r_* = 264.31	*D*_x_ = 1.296 Mg m^−^^3^
Orthorhombic, *P*2_1_2_1_2_1_	Mo *K*α radiation, λ = 0.71073 Å
Hall symbol: P 2ac 2ab	Cell parameters from 14445 reflections
*a* = 6.4210 (14) Å	θ = 2–26.4°
*b* = 13.504 (3) Å	µ = 0.09 mm^−^^1^
*c* = 15.619 (3) Å	*T* = 298 K
*V* = 1354.4 (5) Å^3^	Platelet, colourless
*Z* = 4	0.50 × 0.33 × 0.08 mm

### Data collection

**Table:**

Bruker APEXII CCD area-detector diffractometer	1473 reflections with *I* > 2σ(*I*)
Radiation source: fine-focus sealed tube	*R*_int_ = 0.051
graphite	θ_max_ = 26.4°, θ_min_ = 2.0°
φ and ω scans	*h* = −8→8
14445 measured reflections	*k* = −16→16
1610 independent reflections	*l* = −19→18

### Refinement

**Table:**

Refinement on *F*^2^	Primary atom site location: structure-invariant direct methods
Least-squares matrix: full	Secondary atom site location: difference Fourier map
*R*[*F*^2^ > 2σ(*F*^2^)] = 0.037	Hydrogen site location: inferred from neighbouring sites
*wR*(*F*^2^) = 0.097	H-atom parameters constrained
*S* = 1.08	*w* = 1/[σ^2^(*F*_o_^2^) + (0.0616*P*)^2^ + 0.1199*P*] where *P* = (*F*_o_^2^ + 2*F*_c_^2^)/3
1610 reflections	(Δ/σ)_max_ = 0.002
175 parameters	Δρ_max_ = 0.20 e Å^−^^3^
0 restraints	Δρ_min_ = −0.18 e Å^−^^3^

### Special details

**Table:**

Geometry. All s.u.'s (except the s.u. in the dihedral angle between two l.s. planes) are estimated using the full covariance matrix. The cell s.u.'s are taken into account individually in the estimation of s.u.'s in distances, angles and torsion angles; correlations between s.u.'s in cell parameters are only used when they are defined by crystal symmetry. An approximate (isotropic) treatment of cell s.u.'s is used for estimating s.u.'s involving l.s. planes.

### Fractional atomic coordinates and isotropic or equivalent isotropic displacement parameters (Å^2^)

**Table:**

	*x*	*y*	*z*	*U*_iso_*/*U*_eq_	
C13	0.5094 (4)	0.2383 (2)	0.65504 (15)	0.0622 (7)	
H13A	0.6162	0.2666	0.6231	0.075*	
H13B	0.5024	0.2493	0.7138	0.075*	
C14	0.0186 (5)	−0.14917 (17)	0.73734 (18)	0.0636 (7)	
H14A	−0.0277	−0.2020	0.7042	0.076*	
H14B	0.1063	−0.1606	0.7837	0.076*	
C15	0.0444 (4)	−0.12759 (19)	0.47662 (17)	0.0613 (7)	
H15A	0.0015	−0.1705	0.4309	0.092*	
H15B	0.0570	−0.1652	0.5285	0.092*	
H15C	0.1764	−0.0984	0.4627	0.092*	
C1	−0.1819 (3)	−0.02941 (14)	0.64505 (12)	0.0357 (4)	
H1	−0.2819	0.0185	0.6681	0.043*	
C2	−0.0403 (3)	−0.05775 (15)	0.71848 (12)	0.0398 (5)	
C3	0.0356 (4)	0.02616 (16)	0.77685 (13)	0.0459 (5)	
H3	0.0753	−0.0028	0.8320	0.055*	
C4	0.2238 (4)	0.08094 (17)	0.74103 (12)	0.0434 (5)	
H4A	0.3401	0.0353	0.7378	0.052*	
H4B	0.2619	0.1334	0.7805	0.052*	
C5	0.1881 (3)	0.12596 (13)	0.65264 (11)	0.0318 (4)	
H5	0.0665	0.1697	0.6553	0.038*	
C6	0.1518 (3)	0.04783 (12)	0.58217 (11)	0.0293 (4)	
H6	0.2284	−0.0126	0.5967	0.035*	
C7	−0.0736 (3)	0.02223 (12)	0.56617 (11)	0.0299 (4)	
H7	−0.1476	0.0845	0.5557	0.036*	
C8	−0.1172 (3)	−0.04621 (14)	0.48887 (12)	0.0379 (4)	
C9	−0.3311 (4)	−0.08880 (16)	0.51157 (14)	0.0466 (5)	
H9A	−0.4401	−0.0403	0.5022	0.056*	
H9B	−0.3610	−0.1474	0.4779	0.056*	
C10	−0.3114 (4)	−0.11414 (17)	0.60591 (14)	0.0514 (6)	
H10A	−0.2413	−0.1772	0.6134	0.062*	
H10B	−0.4476	−0.1178	0.6326	0.062*	
C11	0.3685 (3)	0.18293 (14)	0.61722 (12)	0.0369 (4)	
C12	0.3756 (3)	0.16406 (15)	0.52329 (12)	0.0387 (5)	
O1	0.4807 (3)	0.20235 (13)	0.46923 (10)	0.0620 (5)	
O2	−0.1301 (3)	0.00763 (13)	0.40985 (9)	0.0566 (5)	
H2	−0.0522	0.0557	0.4118	0.085*	
O3	0.2403 (2)	0.09088 (10)	0.50419 (7)	0.0362 (3)	
O4	−0.1222 (3)	0.09798 (12)	0.79206 (10)	0.0573 (5)	
H4	−0.2162	0.0730	0.8203	0.086*	

### Atomic displacement parameters (Å^2^)

**Table:**

	*U*^11^	*U*^22^	*U*^33^	*U*^12^	*U*^13^	*U*^23^
C13	0.0621 (16)	0.0839 (17)	0.0406 (11)	−0.0351 (15)	−0.0006 (12)	−0.0034 (12)
C14	0.0740 (17)	0.0480 (12)	0.0688 (16)	−0.0014 (13)	−0.0178 (15)	0.0204 (12)
C15	0.0588 (15)	0.0567 (13)	0.0685 (15)	−0.0020 (13)	0.0073 (13)	−0.0282 (13)
C1	0.0348 (10)	0.0377 (9)	0.0347 (9)	−0.0005 (8)	0.0039 (8)	0.0039 (7)
C2	0.0418 (11)	0.0429 (10)	0.0347 (9)	−0.0039 (9)	0.0016 (9)	0.0100 (8)
C3	0.0584 (13)	0.0511 (11)	0.0283 (8)	−0.0074 (11)	0.0002 (9)	0.0092 (8)
C4	0.0480 (12)	0.0531 (11)	0.0292 (9)	−0.0102 (10)	−0.0060 (9)	0.0037 (9)
C5	0.0326 (9)	0.0346 (8)	0.0283 (8)	−0.0027 (8)	−0.0017 (8)	0.0018 (7)
C6	0.0300 (9)	0.0315 (8)	0.0263 (8)	−0.0003 (7)	−0.0007 (7)	0.0036 (7)
C7	0.0325 (9)	0.0270 (8)	0.0301 (8)	0.0001 (7)	−0.0023 (7)	0.0016 (7)
C8	0.0394 (11)	0.0406 (10)	0.0338 (9)	−0.0087 (9)	−0.0016 (9)	−0.0016 (8)
C9	0.0423 (11)	0.0463 (11)	0.0511 (12)	−0.0115 (10)	−0.0062 (10)	−0.0023 (10)
C10	0.0473 (12)	0.0564 (12)	0.0505 (12)	−0.0189 (11)	−0.0014 (10)	0.0076 (10)
C11	0.0365 (11)	0.0393 (9)	0.0348 (9)	−0.0051 (8)	−0.0011 (8)	0.0024 (8)
C12	0.0385 (11)	0.0425 (10)	0.0350 (9)	−0.0077 (9)	0.0004 (9)	0.0033 (8)
O1	0.0665 (11)	0.0793 (12)	0.0403 (8)	−0.0341 (10)	0.0092 (8)	0.0057 (8)
O2	0.0708 (11)	0.0656 (10)	0.0333 (7)	−0.0277 (9)	−0.0134 (7)	0.0038 (7)
O3	0.0387 (7)	0.0427 (7)	0.0273 (6)	−0.0079 (6)	0.0026 (6)	0.0006 (5)
O4	0.0700 (11)	0.0567 (9)	0.0453 (8)	−0.0069 (9)	0.0244 (9)	−0.0022 (7)

### Geometric parameters (Å, °)

**Table:**

C13—C11	1.314 (3)	C5—C11	1.497 (3)
C13—H13A	0.9300	C5—C6	1.542 (2)
C13—H13B	0.9300	C5—H5	0.9800
C14—C2	1.324 (3)	C6—O3	1.464 (2)
C14—H14A	0.9300	C6—C7	1.509 (3)
C14—H14B	0.9300	C6—H6	0.9800
C15—C8	1.523 (3)	C7—C8	1.546 (2)
C15—H15A	0.9600	C7—H7	0.9800
C15—H15B	0.9600	C8—O2	1.435 (2)
C15—H15C	0.9600	C8—C9	1.530 (3)
C1—C2	1.513 (3)	C9—C10	1.518 (3)
C1—C10	1.541 (3)	C9—H9A	0.9700
C1—C7	1.577 (2)	C9—H9B	0.9700
C1—H1	0.9800	C10—H10A	0.9700
C2—C3	1.534 (3)	C10—H10B	0.9700
C3—O4	1.423 (3)	C11—C12	1.490 (3)
C3—C4	1.523 (3)	C12—O1	1.198 (2)
C3—H3	0.9800	C12—O3	1.349 (2)
C4—C5	1.526 (2)	O2—H2	0.8200
C4—H4A	0.9700	O4—H4	0.8200
C4—H4B	0.9700		
			
C11—C13—H13A	120.0	O3—C6—C7	109.01 (14)
C11—C13—H13B	120.0	O3—C6—C5	105.27 (13)
H13A—C13—H13B	120.0	C7—C6—C5	114.82 (15)
C2—C14—H14A	120.0	O3—C6—H6	109.2
C2—C14—H14B	120.0	C7—C6—H6	109.2
H14A—C14—H14B	120.0	C5—C6—H6	109.2
C8—C15—H15A	109.5	C6—C7—C8	116.10 (15)
C8—C15—H15B	109.5	C6—C7—C1	113.26 (15)
H15A—C15—H15B	109.5	C8—C7—C1	105.42 (14)
C8—C15—H15C	109.5	C6—C7—H7	107.2
H15A—C15—H15C	109.5	C8—C7—H7	107.2
H15B—C15—H15C	109.5	C1—C7—H7	107.2
C2—C1—C10	115.94 (17)	O2—C8—C15	107.27 (18)
C2—C1—C7	116.03 (16)	O2—C8—C9	109.74 (17)
C10—C1—C7	104.85 (15)	C15—C8—C9	111.66 (17)
C2—C1—H1	106.4	O2—C8—C7	112.29 (14)
C10—C1—H1	106.4	C15—C8—C7	113.96 (17)
C7—C1—H1	106.4	C9—C8—C7	101.90 (16)
C14—C2—C1	125.2 (2)	C10—C9—C8	103.59 (18)
C14—C2—C3	117.8 (2)	C10—C9—H9A	111.0
C1—C2—C3	117.04 (16)	C8—C9—H9A	111.0
O4—C3—C4	107.17 (17)	C10—C9—H9B	111.0
O4—C3—C2	112.13 (18)	C8—C9—H9B	111.0
C4—C3—C2	113.11 (17)	H9A—C9—H9B	109.0
O4—C3—H3	108.1	C9—C10—C1	105.22 (17)
C4—C3—H3	108.1	C9—C10—H10A	110.7
C2—C3—H3	108.1	C1—C10—H10A	110.7
C3—C4—C5	113.99 (17)	C9—C10—H10B	110.7
C3—C4—H4A	108.8	C1—C10—H10B	110.7
C5—C4—H4A	108.8	H10A—C10—H10B	108.8
C3—C4—H4B	108.8	C13—C11—C12	121.3 (2)
C5—C4—H4B	108.8	C13—C11—C5	131.25 (19)
H4A—C4—H4B	107.6	C12—C11—C5	107.44 (16)
C11—C5—C4	115.01 (16)	O1—C12—O3	121.53 (18)
C11—C5—C6	101.82 (14)	O1—C12—C11	129.6 (2)
C4—C5—C6	113.31 (15)	O3—C12—C11	108.86 (16)
C11—C5—H5	108.8	C8—O2—H2	109.5
C4—C5—H5	108.8	C12—O3—C6	110.91 (13)
C6—C5—H5	108.8	C3—O4—H4	109.5

### Hydrogen-bond geometry (Å, °)

**Table:**

*D*—H···*A*	*D*—H	H···*A*	*D*···*A*	*D*—H···*A*
O2—H2···O3	0.82	2.42	3.015 (2)	131
O4—H4···O2^i^	0.82	2.03	2.819 (2)	162

Symmetry codes:  (i) −*x*−1/2, −*y*, *z*+1/2.
